# Case Report: A rare mimicker of traumatic brachial plexopathy: intramuscular venous malformation of the subscapularis muscle

**DOI:** 10.3389/fsurg.2026.1943267

**Published:** 2026-09-09

**Authors:** Jin Hang Loh, Zhun Shen Tan, Elaine Zi Fan Soh, Chia Hua Lim, Muhammad Shafiq Azhar, Jamari Sapuan, Shalimar Abdullah

**Affiliations:** 1 Faculty of Medicine, Universiti Kebangsaan Malaysia, Kuala Lumpur, Malaysia; 2 Hand & Microsurgery Unit, Department of Orthopaedics and Traumatology, Faculty of Medicine, Universiti Kebangsaan Malaysia, Kuala Lumpur, Malaysia

**Keywords:** brachial plexopathy, case report, compressive neuropathy, intramuscular venous malformation, sclerotherapy, vascular phleboliths

## Abstract

Venous malformations (VMs) involving the brachial plexus are rare clinical entities that can mimic traumatic brachial plexus injuries. A 27-year-old male presented with progressive right upper limb weakness and numbness following an occupational fall with overhanging limb suggesting a traumatic tractional brachial plexus injury. However, magnetic resonance imaging (MRI) revealed an intramuscular VM in the subscapularis muscle which was compressing the cords of the brachial plexus. In view of difficult surgical access for removal associated with high risks of iatrogenic neurological injury, fluoroscopy-guided percutaneous bleomycin sclerotherapy was performed. Significant neurological recovery was observed post-intervention in conjunction with intensive rehabilitation. This case report outlines the diagnostic clues, management challenges, and functional outcomes of a right subscapularis intramuscular VM causing secondary compressive brachial plexopathy.

## 1 Introduction

Vascular malformations are congenital vascular anomalies that arise from inborn errors of vascular morphogenesis with an overall incidence of 1.5% (1). According to the classification system established by the International Society for the Study of Vascular Anomalies (ISSVA), these lesions represent overabundance of specific types of vessels that are subcategorized by their underlying flow dynamics into fast-flow and slow-flow types (2). This differentiates them from vascular tumors which exhibit abnormal endothelial proliferation and rapid growth postnatally with potential involution; vascular malformations maintain normal endothelial mitotic activity which grow commensurately with the child and never spontaneously regress (3).

Venous malformations (VMs) represent the most common vascular anomaly with an estimated incidence of 1 in 5,000–10,000 births and are typically found in the head and neck, extremities and trunk (4, 5). However, the presence of VMs in the region of brachial plexus causing compressive brachial plexopathy is exceptionally rare. These lesions can remain asymptomatic until significant increase in size or may be associated with the concurrent traumatic event (6). This case report describes the incidental finding of a subscapularis VM that masquerades as post-traumatic brachial plexopathy following an occupational fall and subsequently managed with targeted percutaneous bleomycin sclerotherapy.

## 2 Case report

A timeline summarising the key clinical events is provided in Table 1.

**Table 1:** Timeline of key clinical events.

Time from presentation	Clinical event	Muscle power	Sensation
Month 0	Initial Presentation: Patient was referred to the hand center for right brachial plexus injury evaluation following an occupational fall 3 months ago.	Shoulder abduction: MRC 4 Elbow flexion: MRC 4 Elbow extension: MRC 3 Wrist flexion: MRC 0 Wrist extension: MRC 0 Finger flexion: MRC 0 Finger extension: MRC 0 Finger abduction: MRC 0	Reduced over C5 to T1
Month 1	Subsequent diagnostic investigations: Nerve conduction study and electromyography confirmed subacute right postganglionic pan-brachial plexopathy with evidence of distal continuity. MRI of right brachial plexus: Identified an intramuscular venous malformation in the right subscapularis muscle compressing the posterior and lateral cords. Complementary ultrasound showed minimal intralesional vascularity. CTA showed complete absence of arterial enhancement.		
Month 1.5	Multidisciplinary discussion between hand surgery and interventional radiology. The patient was counseled for sclerotherapy for lesion volume reduction in view of difficult surgical access and potential worsening neurology.		
Month 2.5	First percutaneous sclerotherapy session was performed. A total of 10 mL of 15 mg Bleomycin solution was injected into the right subscapularis lesion under fluoroscopic guidance. Procedure was uneventful and no post-procedural complications noted.		
Month 3.5	Follow up across Orthopaedic and Radiology Clinics. Motor power and sensory examination was similar to the previous presentation. Follow-up Brachial Plexus MRI revealed a significant structural reduction in size measuring 3.3 cm × 6.0 cm × 7.7 cm. A clear fat plane has now emerged between the shrinking subscapularis lesion and the adjacent brachial plexus cords. No perilesional oedema observed. Planned for second sclerotherapy. The patient also underwent regular sessions of physiotherapy and rehabilitation.		
Month 5	Second percutaneous sclerotherapy session was performed. Total of 16 mL of 25 mg bleomycin injected into the right subscapularis lesion under fluoroscopy guidance. Procedure was uneventful and no post-procedural complications noted.		
Month 7	The patient noted improvement of the neurological symptoms. Follow-up Contrasted Brachial Plexus MRI demonstrated the subscapularis lesion is only marginally smaller in its anteroposterior dimension, measuring 2.6 cm × 6.0 cm × 7.7 cm. A clear fat plane is successfully maintained anteriorly between the lesion and the brachial plexus cords. No adjacent muscle oedema seen.	Shoulder abduction: MRC 4 Elbow flexion: MRC 4 Elbow extension: MRC 4 Wrist flexion: MRC 4 Wrist extension: MRC 3 Finger flexion: MRC 3 Finger extension: MRC 0 Finger abduction: MRC 3	Reduced over C6,C7
Month 12	6 months follow up from the last sclerotherapy session. Progressive improvement in neurological symptoms.	Shoulder abduction: MRC 4 Elbow flexion: MRC 5 Elbow extension: MRC 4 Wrist flexion: MRC 4 Wrist extension: MRC 3 Finger flexion: MRC 5 Finger extension: MRC 0 Finger abduction: MRC 3	Remained decreased over C6,C7
2-year	Progressive improvement in neurological symptoms. Patient was able to perform his daily activities.	Shoulder abduction: MRC 4 Elbow flexion: MRC 5 Elbow extension: MRC 5 Wrist flexion: MRC 4 Wrist extension: MRC 4 Finger flexion: MRC 5 Finger extension: MRC 2 Finger abduction: MRC 3	Remained decreased over C6,C7

MRC, Medical Research Council; MRI, magnetic resonance imaging; CTA, computed tomography angiography.

### 2.1 Initial presentation and clinical evaluation

A 27-year-old male with no known medical illnesses or family history of vascular anomalies presented with a 3-month history of progressive right upper limb weakness and numbness (Month 0). The symptoms were preceded after sustaining an occupational fall from height in which he arrested his descent by clinging onto a structure with his right arm. Following his fall, he was initially treated at a local hospital for a presumed traumatic brachial plexus injury before being referred to a specialized hand center due to progressive deterioration. He initially experienced numbness localised to the right radial 3 digits but eventually worsened involving the entire right hand and forearm.

Evaluation of upper limb motor power based on the Medical Research Council (MRC) scale revealed profound deficits in the right upper extremity. Shoulder abduction and elbow flexion were graded as MRC 4, while elbow extension was MRC 3. Complete paralysis (MRC grade 0) for wrist flexion, wrist extension, finger flexion, extension and abduction. Sensation was found to be diminished across the C5 to T1 dermatomes.

**Figure 1:**
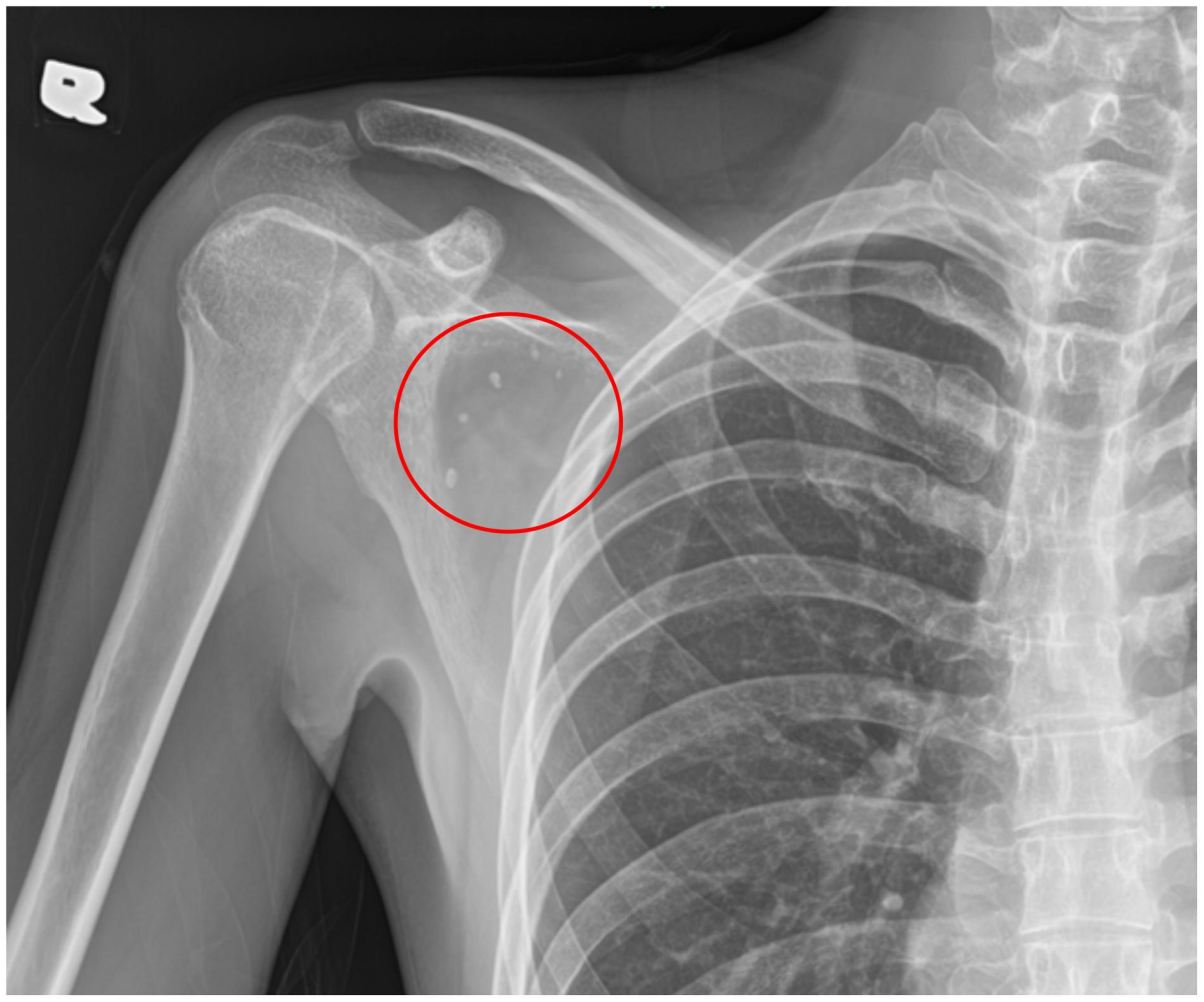
Plain radiograph of the right shoulder demonstrated multiple calcific densities consistent with vascular phleboliths (red circle) suggestive of venous malformations.

**Figure 2:**
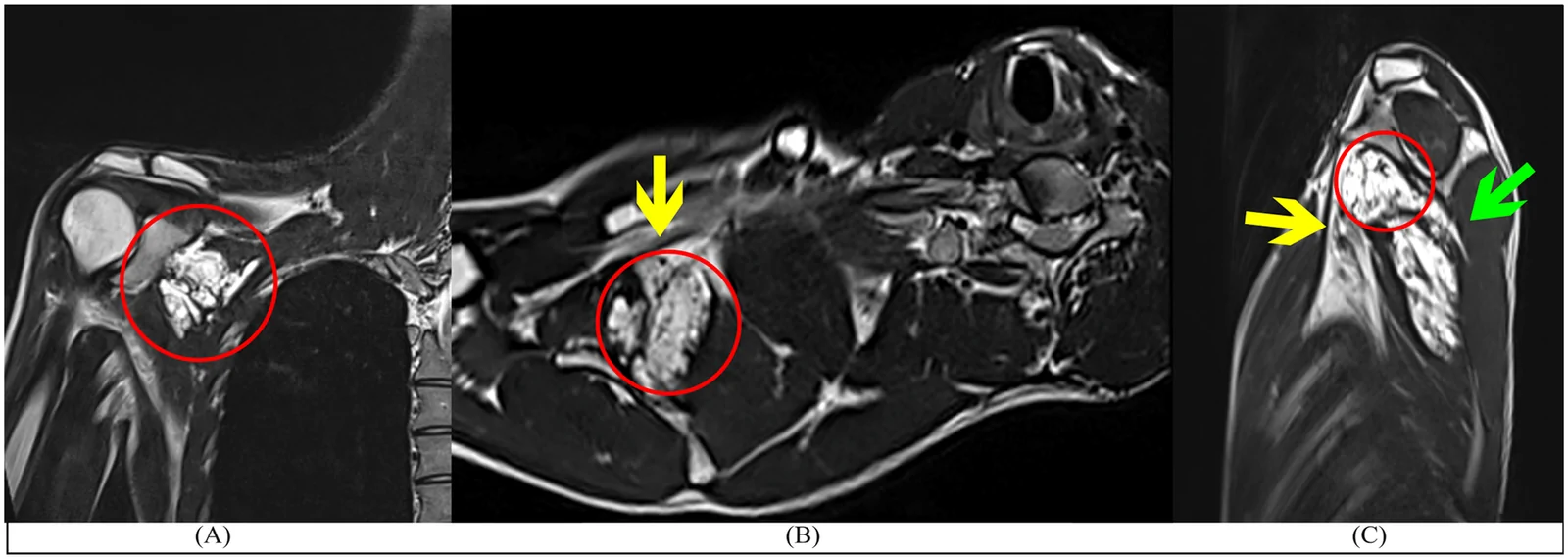
Initial MRI showed a lobulated lesion (red circle) within the right subscapularis muscle measuring 3.7 cm × 6.0 cm × 8.3 cm (AP × W × CC) with hyperintense signal in T2W. The lesion is in close proximity and compressing the cords of the brachial plexus particularly the posterior and lateral cords (yellow arrow). Posteriorly, the lesion is indenting onto the scapula spine (green arrow).

At Month 1, subsequent diagnostic investigations were performed. Plain radiographs of the right shoulder revealed multiple calcific densities suggestive of several phleboliths over the right scapular region (Figure 1). Magnetic resonance imaging (MRI) of the right brachial plexus (Figure 2) revealed a lobulated lesion within the right subscapularis muscle measuring 3.7 cm × 6.0 cm × 8.3 cm (Anteroposterior × Width × Craniocaudal). The lesion demonstrated a slightly hyperintense signal on T1-weighted imaging and an avidly hyperintense signal on T2-weighted imaging. Homogeneous enhancements were seen in the post-contrast sequences. No enlarged feeding artery or draining vein were seen around the lesion. A few rounded, well-defined T1 and T2 hypointense structural foci were seen within the lesion suggestive of phleboliths. The finding of phleboliths was congruent with the findings on plain radiograph. Anteriorly, the lesion was in close proximity with the cords of brachial plexus with possible compression onto the posterior and lateral cords. Posteriorly, it is indenting onto the scapular spine. Otherwise, the roots, trunks, divisions and branches of the brachial plexus are normal in calibre and signal intensity. Overall, the features were suggestive of an intramuscular VM that was compressing the posterior and lateral cords of brachial plexus. Computed tomography angiography (CTA) showed complete absence of arterial enhancement which suggested no features of a high flow lesion.

In view of the previous trauma history, the initial clinical suspicion was favored towards traumatic brachial plexus injury. However, the relevant investigations unmasked the underlying pre-existing vascular lesion which led to the diagnosis of intramuscular low-flow VM of the right subscapularis muscle with secondary compressive brachial plexopathy. If left untreated, there is a risk of permanent neurological deficit due to chronic compression of the lesion. Following a multidisciplinary discussion between the hand surgeons and interventional radiologists, percutaneous sclerotherapy was selected as the preferred therapeutic approach for targeted decompression (Month 1.5). Open surgery resection of subscapularis lesion was considered but avoided in view of difficult surgical access and high risk of iatrogenic neurological deficit. Regular surveillance of the lesion size and neurological symptoms was planned and multiple sessions of sclerotherapy were anticipated depending on the outcome.

### 2.2 Management and clinical progression

**Figure 3:**
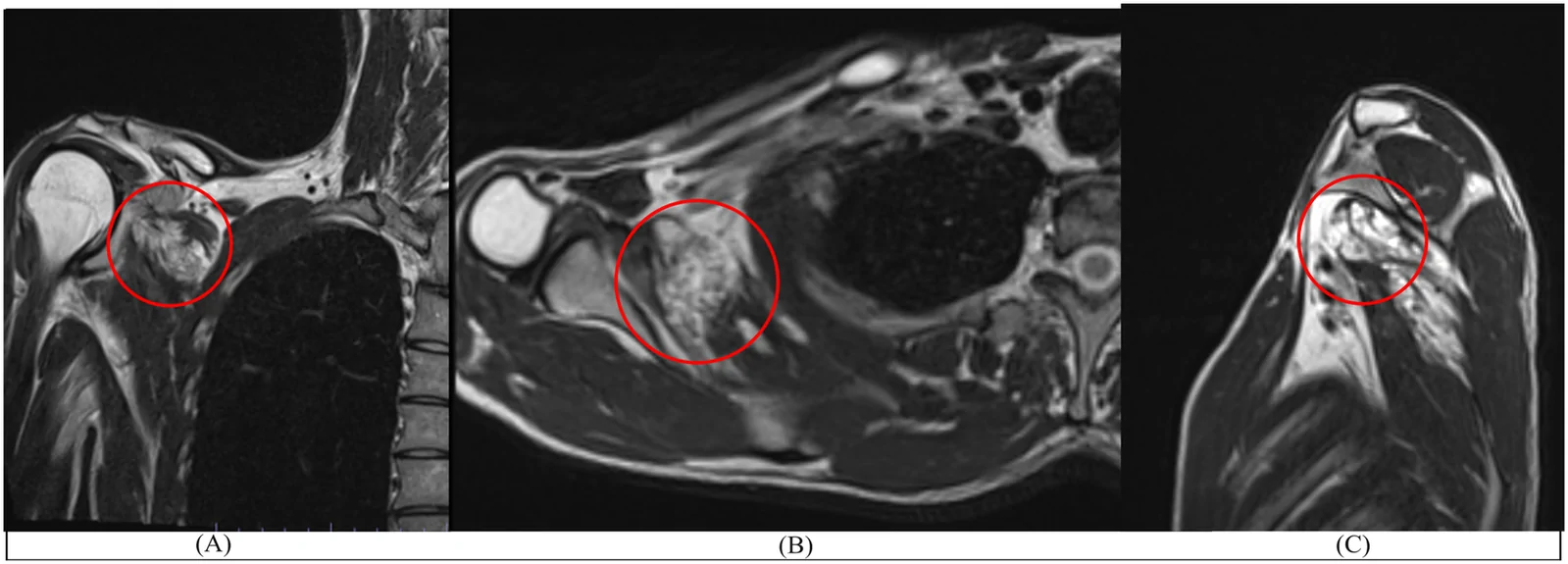
Contrasted MRI (after 2nd sclerotherapy) size of the lesion was marginally smaller 2.6 cm × 6.0 cm × 7.7 cm with clear fat plane seen between the venous malformation and the brachial plexus cords (red circle).

Subsequently, the patient underwent 2 sessions of percutaneous sclerotherapy using bleomycin under fluoroscopy guidance (Month 2.5 to Month 5). Both sessions were well-tolerated with no perioperative adverse events and the patient demonstrated striking neurological recovery. At Month 7, motor power improved significantly in which elbow extension improved to MRC 4, wrist flexion improved to MRC 4, wrist extension improved to MRC 3, finger flexion improved to MRC 3 and finger abduction improved to MRC 3. The other motor powers remained the same. Sensation also improved with remaining deficits localized only to the C6 and C7 dermatomes. On contrasted MRI (Figure 3) after the 2nd sclerotherapy, the lesion size was reduced to 2.6 cm × 6.0 cm × 7.7 cm and the fat planes surrounding the brachial plexus were restored. Following interdisciplinary discussion, further sclerotherapy was deferred due to a plateau in lesion downsizing after the second sclerotherapy sessions and also functional neurological recovery. The patient was also not keen for further sessions nor any surgical procedures including tendon transfer and opted for intensive rehabilitation and regular surveillance. He subsequently received structured rehabilitation at a well-equipped rehabilitation center for 2–3 sessions per week (1 h per session). The interventions performed included transcutaneous electrical nerve stimulation (TENS) therapy, range of motion exercises, strengthening, sensory and motor re-education along with functional training and adaptation with occupational therapy.

At 6 months follow-up (Month 12), the patient reported major functional improvements following intensive rehabilitation and was able to return to work. Surveillance neuroimaging was deferred due to continuous, positive clinical and functional progression. Muscle power shows improvement in which elbow flexion improved to MRC 5 and finger flexion improved to MRC 5. The other motor powers and sensory deficits remained the same. At the latest 2 years follow-up, the patient was completely independent in his daily living and continued to work. Muscle power shows improvement in which elbow extension improved to MRC 5, wrist extension improved to MRC 4 and finger extension improved to MRC 2. The other motor power and sensory deficits remained the same. As part of the long-term management plan, the patient was scheduled for a routine outpatient surveillance MRI and continued physiotherapy.

## 3 Discussion

This case highlights an unique presentation in which the trauma history unmasked an underlying pre-existing vascular lesion. Unlike normal veins, VMs composed of ectatic venous channels and are devoid of a functional smooth muscle layer. This dysmorphic architecture predisposes the lesion to blood stasis and localized intravascular coagulopathy, which leads to spontaneous localized thrombosis that calcifies over time into phleboliths. While VMs often remain asymptomatic while growing commensurately with the patient, a sudden rapid expansion of size can occur following mechanical trauma or hormonal modulation particularly during puberty and pregnancy (6, 7). The temporal relationship between trauma and symptom onset suggests that the trauma may have contributed to lesion expansion or thrombosis; however, a causal relationship cannot be established from this single case.

### 3.1 Radiographic characterization and diagnostic hallmarks of venous malformations

Although plain radiography has limited diagnostic work-up for VMs, it provided the earliest diagnostic clues in this case. The initial plain chest radiograph revealed calcific densities over the right scapular region characteristic of phleboliths. This incidental finding raised clinical suspicion as phleboliths represent pathognomonic radiological hallmarks of VMs due to thrombosis and calcification (7).

MRI remains as the definitive imaging modality for characterising VMs. In this case, the major culprit is revealed on the MRI in which a massive, well defined lobulated right subscapularis lesion consistent with VMs was found compressing on the posterior and lateral cords of brachial plexus. VMs characteristically demonstrate as septated lesions with intermediate to decreased signal intensity on T1-weighted images with increased signal intensity on T2-weighted images. The most definitive diagnostic pathognomonic marker is still the presence of intralesional phleboliths that manifest as distinct low-signal foci across all pulse sequences. On delayed post-contrast T1-weighted imaging, VMs characteristically display diffuse enhancement of the slow-flowing venous channels due to the lack of high flow characteristics such as arterial or early venous phase enhancement, enlarged feeding vessels, and arteriovenous shunting (8, 9).

### 3.2 Sclerotherapy as an option for decompression

Given the challenging surgical access within the subscapular fossa, the multidisciplinary team determined that the primary therapeutic objective should focus on functional decompression of brachial plexus rather than complete eradication of the lesion which may result in iatrogenic neurological injury. To evaluate the safety and suitability of an interventional approach, the lesion was stratified according to the Puig classification system for VMs (10). The previous multi-modal imaging revealed a well-contained intramuscular lesion without macrovascular shunting or dilated ectatic drainage pathways. Hence, the lesion is categorized as a low-risk Type I/II malformation. Sclerotherapeutic intervention in Type I and Type II lesions demonstrate a complication-free success rate of more than 90% due to the absence of dysplastic outflow channels that prevent central embolization of the sclerosing agent. This allows effective concentration of sclerosing agent to remain nearly constant when delivered directly. The well-defined lesion boundaries on MRI imaging along with the absence of noticeable venous drainage represent indicators of favorable results following percutaneous sclerotherapy (8, 11).

Nevertheless, the baseline complication profile of percutaneous sclerotherapy must be acknowledged when high-stakes neural structures lie in direct proximity to the target zone. In a large-scale retrospective review of 573 patients treated with embolo/sclerotherapy for congenital vascular malformations, Lee et al. reported that the baseline incidence of soft tissue injury was 8% specifically within venous-type anomalies, while post-sclerotherapy neuropathy carried an incidence of 10.9%. Despite that, the majority of these complications were reported to recover spontaneously. As a result, embolo/sclerotherapy is suggested as a therapeutic approach for congenital vascular malformations in view of an established acceptable risk- benefit profile when compared to life quality prior to treatment (12).

### 3.3 Sclerosant optimisation

The particular selection of sclerosant fundamentally determines the rates of local and systemic complications. Historically, concentrated absolute ethanol has been the most widely used sclerosant for lesions that cannot be safely accessed via open surgery (9). Ethanol is favored in clinical practice because it is procedurally straightforward, highly effective, inexpensive, readily available, and possesses an extended shelf life. However, the downside is ethanol carries the highest overall complication rate due to its remarkably narrow therapeutic index (7, 13). Cutaneous side effects such as focal skin necrosis, pain, and blistering represent the most common side effects with a reported incidence of 8%. Local ethanol toxicity may lead to peripheral nerve damage in 2%–10% of individuals (11). Beyond localized tissue effects, rare systemic complications can arise if ethanol escapes into systemic circulation. This includes compartment syndrome, arrhythmia, pulmonary embolism, pulmonary hypertension with right heart failure and haemoglobinuria (7, 11).

An alternative sclerosant of choice is bleomycin which is a cytotoxic antineoplastic antibiotic with the lowest complication profiles among other sclerosants (14). Bleomycin is a safer option that causes less intense inflammation which makes it highly preferred over alcohol-based sclerosants in regions that are in close proximity to neural or vascular structure (11, 15). The primary advantage in this case is that it induces significantly less soft-tissue edema and thrombosis which lowers the risk of complications such as compartment syndrome or secondary nerve compression (14).

In terms of volume reduction, bleomycin has proven to be highly effective and functionally non-inferior to absolute ethanol as evidenced by a meta-analysis recording a good-to-excellent volume reduction in 87% of VMs. Crucially, intralesional bleomycin sclerotherapy achieves a vastly superior safety margin with marked reduction in severe regional complications such as full-thickness skin necrosis or permanent peripheral nerve paralysis with 0 documented instances of pulmonary fibrosis encountered across the low-dose interventional registries. The documented local side effects of bleomycin are typically minor and self-limiting which consist primarily of transient local inflammation, localized pain, mild swelling, and minor, reversible skin hyperpigmentation (16). To effectively eliminate the risk of pulmonary fibrosis, strict cumulative threshold monitoring is mandatory with cumulative lifetime dose of bleomycin limited to under 400 mg (or a weight-based ceiling of 5 mg/kg) (9).

### 3.4 Study limitations

The primary limitation of this study is its inherent design as a solitary case report which does not possess the statistical strength necessary to widely generalize these findings to all compressive brachial plexopathies or conclusively determine a relationship between the intervention and the observed neurological recovery. While the patient did not experience severe post-procedural complications such as pulmonary fibrosis, localized tissue necrosis, or an exacerbation of neuropathy, this isolated outcome cannot definitively establish a universal safety profile for the intervention.

Another limitation of this report is the relatively brief post-intervention follow-up of two years which was insufficient to definitively rule out late symptomatic recurrence. In the same study of a retrospective series of 26 patients, a notable recurrence rate of 23% was observed within 2 years across the entire low-flow groups, with the VMs sub-group specifically exhibiting a 20% recurrence rate at 24 months. Despite the limited sample size of the study, the author concluded that post-procedural volume reduction and decompression are achievable but complete long-term remission is extremely uncommon (14). Looking back at our case, the inability to draw definitive prognostic conclusions is further limited by a profound lack of statistical data specific to brachial plexus lesions in the current literature.

It must also be acknowledged that percutaneous sclerotherapy alone is insufficient to achieve optimal outcomes. The success of our case relies heavily on a synergistic combination with intensive physical rehabilitation. Even with dual-modality management, complete functional restoration is never guaranteed, and patients may still eventually require secondary reconstructive optimization via tendon transfers to address minor residual deficits. Despite the high efficacy and favorable safety profile of percutaneous bleomycin sclerotherapy, the risk of recurrence necessitates long-term outpatient MRI surveillance.

## 4 Conclusion

In conclusion, this case highlights a rare presentation of a compressive subscapularis intramuscular VM that was initially masqueraded as traumatic plexopathy. The primary diagnostic takeaway emphasizes that the pathognomonic discovery of phleboliths on plain radiographs must prompt immediate MRI to evaluate for any possible underlying vascular anomaly to prevent any diagnostic delay or unnecessary surgical exploration. This case demands the coordination of a multidisciplinary team among hand surgeons, interventional radiologists, and rehabilitation specialists to deliver the most optimal outcome. Ultimately, this case demonstrates that sclerotherapy serves as a viable alternative for VMs with difficult surgical access. Nevertheless, rather than focusing solely on volumetric radiographic eradication, the main therapeutic goal should shift to alleviate patient-reported symptoms and enhancing overall quality of life through long-term monitoring.

## Data Availability

The original contributions presented in the study are included in the article/Supplementary Material, further inquiries can be directed to the corresponding author.
